# Rare-Earth-Doped Calcium Carbonate Exposed to X-ray Irradiation to Induce Reactive Oxygen Species for Tumor Treatment

**DOI:** 10.3390/ijms20051148

**Published:** 2019-03-06

**Authors:** Chun-Chen Yang, Wei-Yun Wang, Feng-Huei Lin, Chun-Han Hou

**Affiliations:** 1 Department of Materials Science and Engineering, National Taiwan University, Taipei 10617, Taiwan; d03527007@ntu.edu.tw (C.-C.Y.); smallcloud929@hotmail.com.tw (W.-Y.W.); 2 Institute of Biomedical Engineering, National Taiwan University, Taipei 10617, Taiwan; 3 Institute of Biomedical Engineering and Nanomedicine, National Health Research Institutes, Miaoli 35053, Taiwan; 4 Department of Orthopedic Surgery, National Taiwan University, Taipei 10617, Taiwan

**Keywords:** calcium carbonate, cerium, X-ray, alternative tumor treatment

## Abstract

Conventional photodynamic therapy (PDT) is limited by its penetration depth due to the photosensitizer and light source. In this study, we developed X-ray induced photodynamic therapy that applied X-ray as the light source to activate Ce-doped CaCO_3_ (CaCO_3_:Ce) to generate an intracellular reactive oxygen species (ROS) for killing cancer cells. The A549 cell line was used as the in vitro and in vivo model to evaluate the efficacy of X-ray-induced CaCO_3_:Ce. The cell viability significantly decreased and cell cytotoxicity obviously increased with CaCO_3_:Ce exposure under X-ray irradiation, which is less harmful than radiotherapy in tumor treatment. CaCO_3_:Ce produced significant ROS under X-ray irradiation and promoted A549 cancer cell death. CaCO_3_:Ce can enhance the efficacy of X-ray induced PDT, and tumor growth was inhibited in vivo. The blood analysis and hematoxylin and eosin stain (H&E) stain fully supported the safety of the treatment. The mechanisms underlying ROS and CO_2_ generation by CaCO_3_:Ce activated by X-ray irradiation to induce cell toxicity, thereby inhibiting tumor growth, is discussed. These findings and advances are of great importance in providing a novel therapeutic approach as an alternative tumor treatment.

## 1. Introduction

Photodynamic therapy (PDT), a common clinical treatment for tumors, uses light of specific wavelengths to activate photosensitizers (PS) to destroy or damage cells by the generation of reactive oxygen species (ROS) [1,2,3]. The excited PS can generate cytotoxic ROS, such as peroxides (·O_2_^2−^), superoxide ions (·O_2_^−^), hydroxyl radicals (·OH), and singlet oxygen (^1^O_2_) through type I and type II reactions, which mediate cell injury by peroxidising lipids or inactivating proteins leading to necrosis or apoptosis [4,5,6]. The oxidative power of a vast selection of photo-catalysts has been assessed and relevant operating parameters for the photo-degradation of methylene blue (MB) have been also investigated in a previous study [7].

Most clinical applications use light in the wavelength range of 630–800 nm to achieve the deepest tissue penetration, and several clinical photosensitizers available have significant absorption bands in this region [8]. However, the effective depth of treatment is typically less than 1 cm, so that optimal light delivery to deep-seated or larger tumors or alternative nanoparticle strategies may be required [9,10].

Ionizing radiation, such as X-rays or gamma rays, could be alternative light sources that enable deeper penetration (8–14 cm) in tissues with optimized sensitizers [11,12]. X-rays may interact with molecules to generate free radicals and ROS, which can damage DNA or cellular organelles [13,14,15]. Therefore, X-rays could be used as alternative light sources to achieve a deeper penetration depth and are less dangerous and hazardous than gamma rays [14].

**Figure 1:**
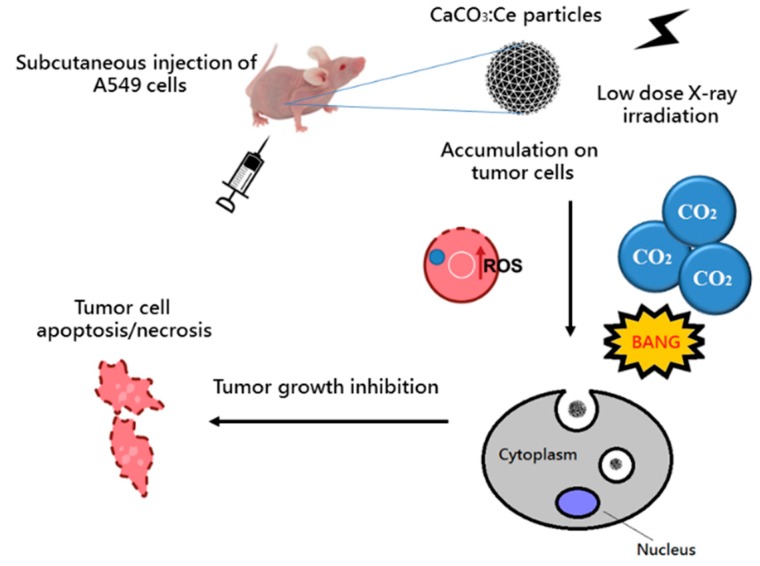
The mechanism of CaCO_3_:Ce activated by X-ray to generate ROS and CO_2_ for tumor growth inhibition.

Calcium carbonate (CaCO_3_) has been used in drug delivery systems because of its great biocompatibility, low toxicity, easy production and slow biodegradability [16,17,18]. Principally, CaCO_3_ has three crystalline phases, namely calcite, vaterite and aragonite. Aragonite has an orthorhombic structure, usually found as needle-like particles, while calcite has a rhombohedral structure, often shaped as cubes [19]. Vaterite has a hexagonal structure that usually results in spherulitic particles and is rarely found in nature as it easily and irreversibly transforms into a more thermodynamically stable phase when in contact with water. Cerium (atomic number = 58) is a non-toxic rare-earth element and has previously been proven to enhance photochemical reactions owing to its strong catalytic potential and light response extension [20,21]. It can also suppress the recombination of electron–hole pairs and extend their lifetime. In addition, cerium has a larger X-ray photon interaction cross-section than that of biological tissue constituents, which leads to intense X-ray interaction with host materials and consecutive ROS generation [22]. The entire process is illustrated in Figure 1.

The specific aim of this study was to synthesize CaCO_3_:Ce phosphor that can be activated by X-ray for photodynamic therapy, which can be used as an adjuvant therapy for cancer and lower the radiation dose.

X-ray diffraction (XRD), field-emission scanning electron microscopy (FESEM), and energy dispersive X-ray spectroscopy (EDX) were performed for crystal structure identification, surface morphology observation, and composition analysis, respectively. The degradation of methylene blue (MB) was evaluated to examine the generation of ROS. The adenocarcinoma human alveolar basal epithelial cell line (A549) was used for in vitro and in vivo studies to evaluate the effect of the synthesized CaCO_3_:Ce in X-ray-induced PDT. The in vitro testing of the synthesized CaCO_3_:Ce in X-ray-induced PDT was analyzed by lactate dehydrogenase (LDH) assay and then further assessed by live/dead staining. Tumor-induced BALB/c nude mice were used to check the efficacy and efficiency of the CaCO_3_:Ce as the photosensitizer in in vivo PDT treatment. A549 cells were injected into mouse thighs subcutaneously, to develop tumor lesions for use as an animal model. The tumor size was measured to examine the therapeutic result of the CaCO_3_:Ce in X-ray-induced PDT. The blood/serum analysis and hematoxylin and eosin stain (H&E) examination were used for safety assessment of the mice. The results and findings in the study were collected and further analyzed to prove that the developed CaCO_3_:Ce could be the most promising photosensitizer in X-ray-induced PDT for clinical use.

## 2. Results

**Figure 2:**
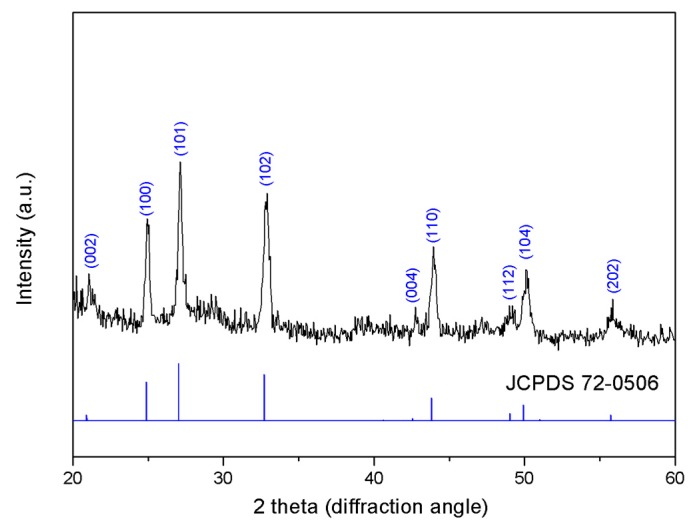
X-ray powder diffraction pattern of CaCO_3_:Ce by Cu Kα radiation (λ = 1.5406 Å) in 2θ ranging from 20° to 60°.

The XRD pattern of CaCO_3_:Ce particles is presented in Figure 2. The indexed peaks correspond to the hexagonal vaterite (JCPDS No. 72-0506), which are positioned at 2θ values of 21.1°, 25.0°, 27.1°, 32.8°, 42.7°, 43.9°, 49.0°, 50.2° and 55.7°, corresponding to (101), (004), (112), (200), (105), and (211), respectively.

**Figure 3:**
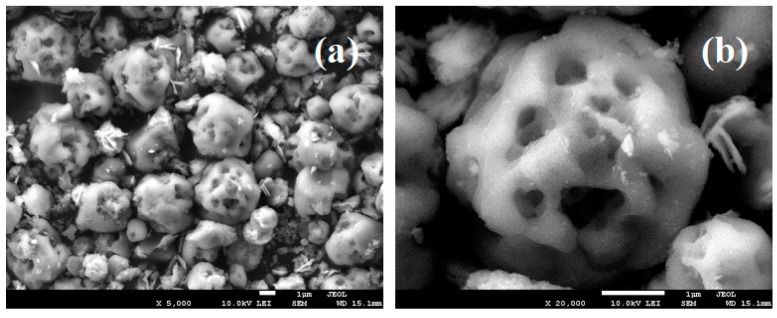
SEM images of CaCO_3_:Ce particles at the (**a**) low magnification; (**b**) high magnification.

Figure 3 shows the SEM images of the CaCO_3_:Ce particles. It is evident that the spherical particles have a size of approximately 1–3 μm with uniform distribution from the low magnification image. From the high magnification view, the isolated micro-pores were found to have an average size of about 500 nm. The results of Brunauer–Emmett–Teller (BET) test was shown in Table S1.

**Figure 4:**
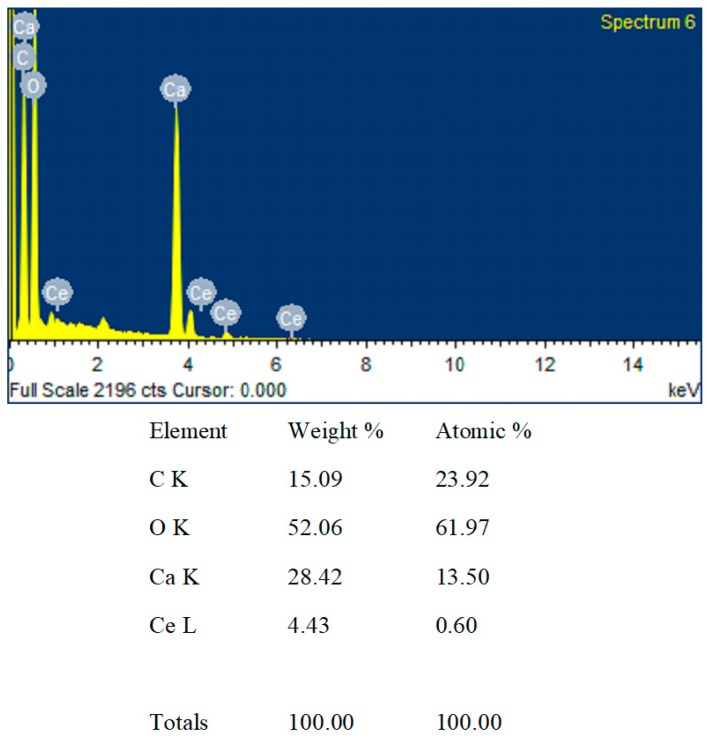
Spectra and chemical composition chart of CaCO_3_:Ce particles.

Figure 4 shows the chemical composition analysis of CaCO_3_:Ce by EDX, one of the accessories of the SEM. Ca, C, O, and Ce elements were detected. There was no trace of any other elements or contaminants within the detection limit of the EDX. The atomic ratio of cerium to calcium was 1:22.5.

**Figure 5:**
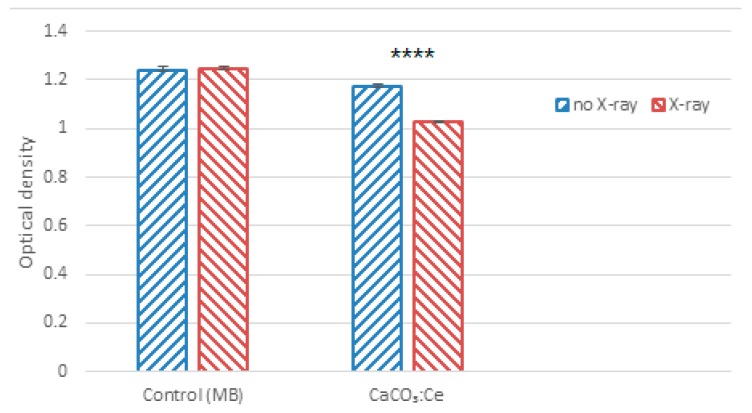
The generation of ROS of CaCO_3_:Ce particles under X-ray irradiation determined by the degradation of methylene blue (*t*-test, mean ± SD, *n* = 6, ****: *p*-value < 0.0001).

Figure 5 shows that there is no difference between 10 ppm MB solution without and with X-ray irradiation. When 10 mg CaCO_3_:Ce particles were added to the MB solution and exposed to X-rays, MB was degraded by up to 14.37% compared to without X-ray exposure. This result indicated the significant generation of ROS from CaCO_3_:Ce when exposed to X-ray irradiation.

**Figure 6:**
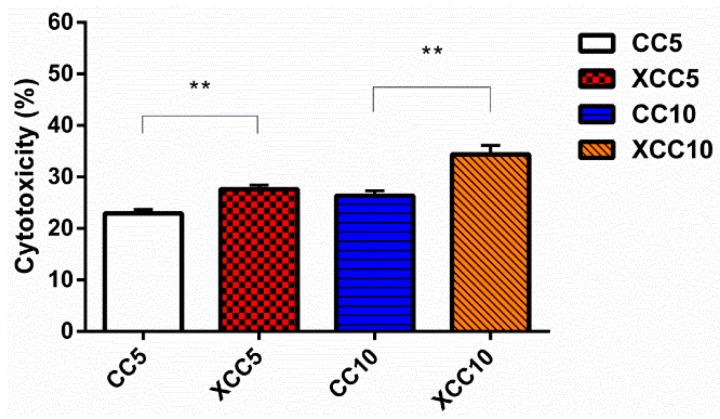
Cell cytotoxicity induced by CaCO_3_:Ce under X-ray irradiation (*t*-test, mean ± SD, *n* = 6, **: *p*-value < 0.01).

The in vitro PDT effects of CaCO_3_:Ce activated by X-ray irradiation were evaluated using LDH assay with A549 lung cancer cells (Figure 6). At day 3, the death rate of A549 cells in XCC5 was 27.65 ± 0.79%. Cytotoxicity in CC10 at day 3 was at acceptable levels of 26.35 ± 0.99%. In contrast, cytotoxicity in the XCC10 group was 34.36 ± 1.77%, which indicated the cellular death caused by CaCO_3_:Ce after exposure to X-ray irradiation. 

**Figure 7:**
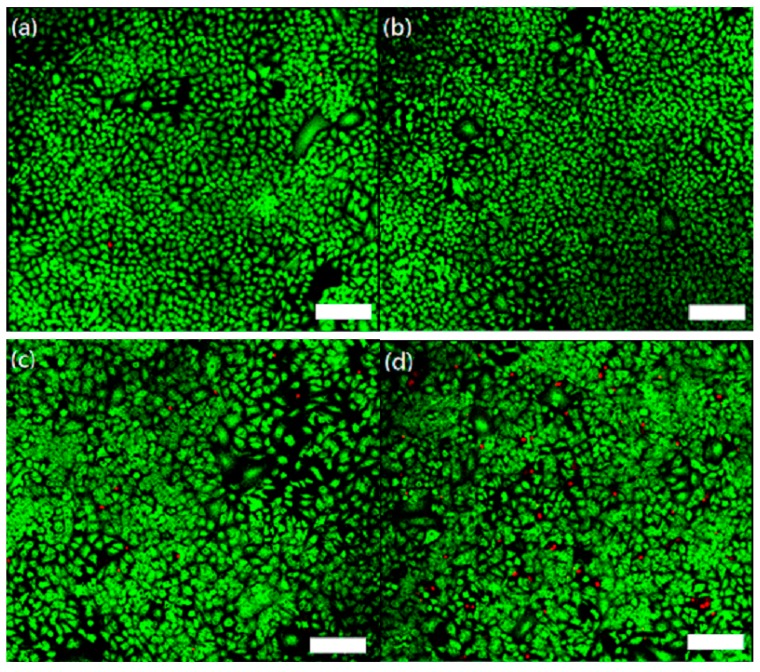
The PDT efficacy of CaCO_3_:Ce exposed to X-ray irradiation, evaluated by live/dead staining of (**a**) control group; (**b**) X-ray-treated group; (**c**) CaCO_3_:Ce group; and (**d**) PDT-CaCO_3_:Ce group, where living cells produced green fluorescence and dead cells showed red fluorescence (scale bars: 200 μm).

The results of live/dead assay are shown in Figure 7 to evaluate cell viability of CaCO_3_:Ce under X-ray irradiation. At day 3, a large number of live A549 cells (green fluorescence) were found in the control group (Figure 7a). Both the X-ray treated group (Figure 7b) and CaCO_3_:Ce group (Figure 7c) show very few dead cells (red fluorescence), which was in agreement with the biocompatibility results. In Figure 7d a large number of dead cells following CaCO_3_:Ce after being activated by X-ray irradiation can be seen, indicating the decrease in cell viability and cellular death.

**Figure 8:**
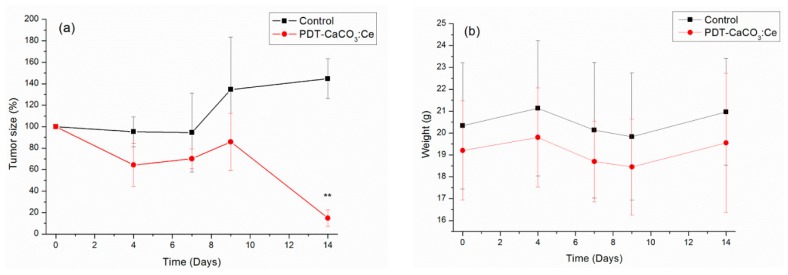
The tumor size (**a**) and body weight (**b**) in BALB/c nude mice injected with CaCO_3_:Ce and subjected to X-ray irradiation (*t*-test, mean ± SD, *n* = 4, **: *p*-value < 0.01).

After the effect of CaCO_3_:Ce under X-ray irradiation was proven to kill A549 cancer cells in vitro, an in vivo study was carried out to prove the PDT effect as well (Figure 8). Due to the consideration of the cell killing effect and to reduce the use of animals, we chose CaCO_3_:Ce under X-ray irradiation, indicated as PDT-CaCO_3_:Ce, as the main experimental group. The anti-tumor efficacy of the PDT-CaCO_3_:Ce group was evaluated by the change in tumor size in male BALB/c nude mice. Figure 8a shows that the tumor volume of the control group at day 14 had increased up to 140% compared to the initial volume. In contrast, on day 14, the tumor volume of the PDT-CaCO_3_:Ce group sharply decreased to nearly 15% of the initial volume. The results indicated that CaCO_3_:Ce could be a promising photosensitizer activated by X-ray to great anti-tumor effect.

The safety efficacy of PDT-CaCO_3_:Ce was evaluated by whole blood/serum analysis and H&E examination. From the blood/serum biochemical analysis (Table 1), it was found that all parameters of the PDT-CaCO_3_:Ce group complied with the standard and were in the range of the standards of blood biochemical analysis (Table 2). The heart, lung, liver, kidney, and spleen were harvested from the mice to determine the toxicity that could arise from the effect of CaCO_3_:Ce after X-ray irradiation.

**Figure 9:**
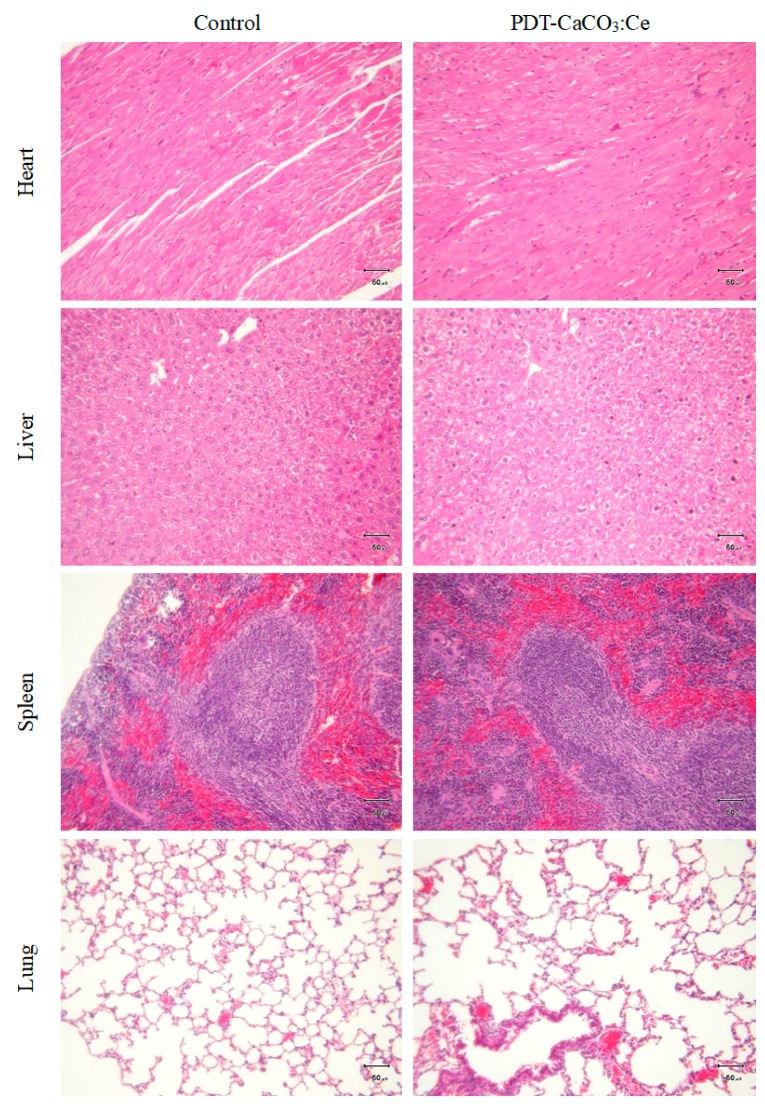
H&E-stained slices of major organs harvested from scarified BALB/c nude mice at the end of the animal study and examined under optical microscope (scale bar: 50 μm).

Figure 9 shows no significant difference between the experimental group and control group, including no observable signs of side effects on normal tissues from H&E staining.

## 3. Discussion

The option of using photosensitizers and light sources to improve the shallow tissue penetration depth has been garnering significant interest as a key element of an alternative therapeutic modality. The size, shape and structure of a material, which relate to toxicity, are viewed as an important determination for use as a photosensitizer, so it is necessary to characterize materials before cell viability and cell cytotoxicity tests [23,24,25]. 

CaCO_3_:Ce was successfully synthesized using the co-precipitation method and confirmed by crystalline phase identification, as shown in Figure 2. The CaCO_3_:Ce particle was spherical with a diameter of 1–3 μm. The surface of the particles was very rough and consisted of a large number of pores and channels (Figure 3). Large particles are most likely to be engulfed via micropinocytosis [26]. The spherical and rough particles will have a larger surface area, which may cause higher reactivity with nearby particles, resulting in possibly harmful effects, such as oxidative stress, ROS generation, and mitochondrial perturbation [27,28]. The biological identity that could influence ist interactions with the cells was further observed in the cell viability and cell cytotoxicity tests. X-ray was used as the alternative light source to activate the photosensitizer in this study. The results indicated that the degradation of MB in the absence of X-ray would greatly extend the range of applications for CaCO_3_-based degradation processes, which is evident that the oxidative behavior is due to the generation of ROS at the catalyst surface (Figure 5). The increase in ROS caused by exposure to X-ray irradiation may be attributed to the oxygen enhancement of radiolysis by ionizing radiation [29,30]. Cerium provides a larger photon interaction cross-section to absorb high-energy X-ray irradiation, which induces the excitation and ionization of water molecules, leading to the overproduction of ROS [22,31]. It is suggested that when the antioxidant defenses are overwhelmed, the overproduction of ROS in cancer cells, which leads to oxidative damage to the proteins, DNA, and lipids in mitochondria may cause mitochondrial dysfunction [32,33]. Briefly, when the increase in ROS reaches the toxic threshold, cell death will be triggered, as can be seen in Figure 6. Different pH values existed in the microenvironment of the tumor tissues (pH 6.5–6.8), cell endosomes (pH 5.5–6.5), lysosomes (pH 4.5–5.5), blood stream and normal tissues (pH 7.4) [34,35]. CaCO_3_ itself is known to react with protons and dissolve to generate CO_2_ gas in an acidic environment, as described in Equations (1) to (4) [36].

CaCO_3(s)_ → Ca^2+^ + CO_3_^2−^ (1)

CO_3_^2−^ + H^+^ → HCO_3_^−^ (2)

HCO_3_^−^ + H^+^ → H_2_CO_3_ (3)

H_2_CO_3_ → H_2_O + CO_2_ (4)

Previous studies show that CO_2_ affects the expression of intracellular messengers that relate to cell death, such as PGC-1a, TFAM, the protein levels of cleavage products of caspase-3, caspase-9, and PARP [37]. CO_2_ also significantly inhibits tumor growth, tumor new blood vessel formation, and cancer cell invasion by affecting the expression of hypoxia-inducible factor 1-alpha (HIF-1a), vascular endothelial growth factor (VEGF), matrix metalloproteinase-2 (MMP-2), and matrix metalloproteinase-9 (MMP-9) [37,38]. The mechanism of this was that CaCO_3_:Ce would be identified as a foreign body by cells, which stimulates a foreign body reaction associated with the activation of neutrophils (also known as polymorphonuclear leukocytes, PMNs). The activation of neutrophils promotes the assembly of nicotinamide adenine dinucleotide phosphate (NADPH) oxidase subunits at phagosome and/or plasma membranes [39]. NADPH oxidases are integral membrane proteins that transfer electrons from NADPH to oxygen across biological membranes, generating O_2_^−^ and H_2_O_2_ [40]. In the endosome/lysosome, the pH value was as low as 3–4, which may cause CaCO_3_ to dissolve in the confined space and produce CO_2_, which has a bombing effect that further causes cell death. ROS also react with membranes and can oxidize membrane proteins, cholesterol and polyunsaturated lipids in a process called lipid peroxidation, resulting in cell death [41]. After CaCO_3_:Ce excitation by X-ray, causing oxidative damage and a decrease in cell viability that is related to either intracellular component leakage or Ce entrance into the damaged cells, and the direct attack of nuclei and other intracellular components (Figure 7). On the other hand, CaCO_3_:Ce could react in an acidic tumor environment to generate CO_2_. The small CO_2_ bubbles hiding in porous channels of CaCO_3_:Ce would burst after exposure to X-ray, resulting in inertial cavitation. These CO_2_ bubbles will instantly explode to destroy tumor tissues and vessels once exposed to X-ray, and consequently occlude the blood supply within the tumor, inducing cell necrosis. The observations of in vivo tumor size (Figure 8) indicated that CaCO_3_:Ce activated by X-ray could be an effective method to cause evident cell death and inhibit tumor growth. CaCO_3_:Ce is also non-toxic without exposure to X-ray. It possesses great biocompatibility, low toxicity, causing no side effects to normal tissues after treatment (Figure 8). CaCO_3_:Ce activated by X-ray could be a promising approach for further adjunctive tumor treatment.

## 4. Materials and Methods

### 4.1. Preparation of CaCO_3_:Ce

Briefly, 0.53 g of sodium carbonate (Na_2_CO_3_, Sigma-Aldrich, St. Louis, MO, USA) was dissolved in 50 mL ddH_2_O to make 0.1 M Na_2_CO_3_ solution. Secondly, 40 g/L of PVA (Sigma-Aldrich) and 0.618 mL Tween 80 (Sigma-Aldrich) were mixed with Na_2_CO_3_ solution, and then added into 50 mL ddH_2_O as Solution A. After that, 1.18 g of calcium nitrate tetrahydrate (Ca(NO_3_)_2_·4H_2_O, Sigma-Aldrich) was dissolved in 50 mL ddH_2_O to make 0.1 M Ca(NO_3_)_2_·4H_2_O solution. Next, 0.217 g of Cerium(III) nitrate hexahydrate (Ce(NO_3_)_3_·6H_2_O, Alfa, Ward Hill, MA, USA) was mixed with Ca(NO_3_)_2_·4H_2_O solution, and then added into 50 mL ddH_2_O as Solution B. Afterwards, Solution B was rapidly poured into Solution A and stirred at room temperature for 1 h to obtain the mixture. The mixture was added drop wise to 100 mL hexane (Sigma-Aldrich), and then rinsed with ethanol. The sample was dried in an oven and calcined at 325 °C for 2 h.

### 4.2. Material Characterization

The crystalline phase of the synthesized CaCO_3_:Ce was determined by X-ray diffractometry (XRD; Philips, 1830/Mac, Amsterdam, The Netherlands) with CuKα radiation (λ = 0.15406 nm) at 30 kV and 15 mA. The diffraction patterns were collected at a scanning speed of 2°/min from 20° to 60°. The surface morphologies were observed with a field-emission scanning electron microscope (FESEM; JEOL, JSM-7600F, Akishima, Tokyo, Japan) at 10 kV and 30 mA. The chemical composition of the samples was analyzed with energy-dispersive X-ray spectroscopy (EDX; JEOL, JSM-7600F, Akishima, Tokyo, Japan). 

### 4.3. Degradation of MB for ROS Detection

In order to evaluate ROS production of the synthesized CaCO_3_:Ce under X-ray irradiation, the degradation of MB (Sigma-Aldrich) was analyzed by an enzyme-linked immunosorbent assay (ELISA, Thermo Scientific, Waltham, MA, USA) reader at an absorbance of 664 nm. X-ray apparatus (POYE, PX-80M, New Taipei, Taiwan) at 80 kV and 10 mA was used to generate X-ray at a dose rate of 0.08 Gy/min for 100 s. Ten micrograms of CaCO_3_:Ce was dispersed in 10 mL of aqueous solution of 10 ppm MB. Ten ppm MB with no particles was viewed as the control group. 

### 4.4. LDH assay: In Vitro Testing of Synthesized CaCO_3_:Ce in PDT 

The effects of CaCO_3_:Ce in PDT were analyzed by LDH assay (TaKaRa, Kusatsu, Shiga, Japan) to assess cell death rate, respectively. A549 cells were seeded on a 96-well plate at a density of 3 × 10^3^ cells/well and then incubated at 37 °C in a 5% CO_2_ atmosphere. One day after seeding, 100 μL of 0.5 mg/mL and 1 mg/mL of synthesized particles were added to each well without X-ray irradiation, representing CC5 and CC10, respectively. The XCC5 and XCC10 stand for cells cultured with CaCO_3_:Ce in the concentration of 0.5 mg/mL and 1 mg/mL and subjected to X-ray radiation after incubating for 4 h. The radiation parameters of the X-ray apparatus were described in Section 4.3. The cultured medium was collected for the LDH assay; 50 μL of cultured medium was mixed and reacted with 50 μL of LDH reagent for 30 min in the dark at room temperature. After the reaction, 50 μL of stop solution was added to each well to stop the reaction, and then the cell death rate was evaluated by using a microplate reader at the absorbance of 490 nm. F12K medium (Sigma-Aldrich) supplemented with 10% Fetal Bovine Serum (FBS, Gibco, Waltham, MA, USA) and 1% antibiotic-antimycotic (Gibco) was used for A549 cell culture. Each experiment was repeated 6 times.

### 4.5. Live/Dead Staining: In Vitro Testing of Synthesized CaCO_3_:Ce in PDT

The live/dead staining kit contains two components. A dye calcein AM (Thermo Fisher) that permeates the plasma membrane and when cleaved by intracellular esterases fluoresces green and can be used to identify live cells. The second dye, ethidium homodimer–1 (Thermo Fisher), exhibits red fluorescence when intercalated into the DNA of dead or damaged cells. A549 cells (BCRC) were seeded in a 3.5 cm culture dish (Thermo Fisher) at a density of 8.5 × 10^4^ cells/dish and cultured for one day. The synthesized CaCO_3_:Ce (1 mg/mL) in 2 mL of medium was added to each culture dish. After a 4 h reaction time, cells were irradiated with X-ray generated at 80 kV and 10 mA at a dose rate of 0.08 Gy/min for 100 s. All dishes were incubated at 37 °C in a 5% CO_2_ atmosphere for one day. In the following two days, l mL of live/dead reagent prepared with phosphate-buffered saline (PBS, Thermo Fisher) was added into each dish and kept in the dark for 30 min, and then observed with a confocal microscope.

### 4.6. Tumor Observation: In Vivo Evaluation of Synthesized CaCO_3_:Ce in PDT 

A549 cells at a density of 10^6^ cells/100 μL suspended in PBS were subcutaneously injected into the thighs of seven-week-old male BALB/c nude mice, which were maintained in cages by normal feeding for 2 weeks until tumors grew to a volume of 25 mm^3^. The tumor volume was calculated using a Vernier caliper with the following formula: *v* = 0.5*ab*^2^ (where *a* is the long diameter of the tumor, *b* is the short diameter of the tumor, and *v* is volume). The mice were divided into two groups: (1) the control group was injected with only PBS at day 0 and day 7 without X-ray irradiation; and (2) the experimental group, abbreviated as PDT-CaCO_3_:Ce, had an intratumoral injection of CaCO_3_:Ce particles at day 0 and day 7, and were exposed to X-ray irradiation at day 0, day 4, day 7 and day 9. The whole experiment period from tumor growth until mice being sacrificed lasts for 4 weeks.

### 4.7. Blood/Serum Analysis and Histological Examination: In Vivo Safety Testing of Synthesized CaCO_3_:Ce in PDT 

After all the mice were sacrificed, the whole blood, serum sample and internal organs were harvested for later analysis and examination as follows. Major organs were harvested and fixed with 10% formaldehyde. Fixed tissues were embedded in paraffin, sectioned into slices with a thickness of 5 μm, and stained with hematoxylin and eosin (H&E) for histological examination. Whole blood was collected using 23G needles through cardiac puncture without thoracotomy, sampled into a tube with 7.5% Ethylenediaminetetraacetic acid (EDTA) solution, and sent to the Animal Center of National Taiwan University for further analysis. Blood/serum biochemical analysis includes renal function testing, liver function testing, and electrolyte examination. Blood urine nitrogen (BUN) and creatinine (CREA) were used for renal function examination. Alanine aminotransferase (ALT), aspartate aminotransferase (AST), and albumin (ALB) are used for evaluation of liver function. Calcium (Ca) and inorganic phosphate (IP) were measured as an assessment of electrolytes. Blood biochemical analysis comprised blood routine and white blood cell classification. All of the animal experiments were executed according to the principles of the 3Rs—reduction, refinement, and replacement of animal use in research—supervised by the Animal Center of National Taiwan University for Animal Welfare. 

### 4.8. Statistical Analysis

All of the data were collected and expressed as mean ± standard deviation (SD) for the control and experimental groups. Statistical analysis was performed in GraphPad Prism 6 and analyzed by *t*-test in this study. The *p* value < 0.05 was considered statistically significant.

## 5. Conclusions

We developed CaCO_3_:Ce activated by X-ray as a potent approach to produce large amounts of ROS applied in adjunctive tumor treatment. The CaCO_3_:Ce particle was synthesized by the co-precipitation method and confirmed by crystalline phase identification in the vaterite phase. The cell viability and cytotoxicity of CaCO_3_:Ce evaluated by WST-1 and LDH assays showed no intracellular cytotoxicity to normal cells compared to the control group. The cytotoxicity increased when CaCO_3_:Ce was activated by X-ray, and cell death was induced via necrosis or apoptosis in human lung adenocarcinoma A549 cells. The result was consistent with ROS detection by the degradation of MB. The in vivo study also showed tumor growth inhibition based on the cell death mechanism induced by the overproduction of ROS and CO_2_. A safety assessment by blood/serum analysis and H&E examination indicated that CaCO_3_:Ce is not toxic and causes no side effects to normal tissues. This study provides a novel therapeutic approach as an alternative tumor treatment.

## Figures and Tables

**Table 1:** Bio-chemical analysis of blood samples from PDT-CaCO_3_:Ce group.

Analysis	Standard	PDT-CaCO_3_:Ce Group
RBC (M/μL)	7.42–9.86	9.46
Hb (g/dL)	12.70–18.40	15.00
HCT (%)	35.44–47.16	46.40
MCV (fL)	45.74–49.86	49.00
MCH (pg)	13.30–16.50	15.90
MCHC (g/dL)	29.09–36.29	32.30
WBC (K/μL)	2.19–3.63	3.11
LYMPH (K/μL)	0.15–0.87	1.12
MONO (K/μL)	0.09–0.53	0.17

**Table 2:** Bio-chemical analysis of the blood/serum samples from PDT-CaCO_3_:Ce group.

Analysis	Standard	PDT-CaCO_3_:Ce Group
BUN (mg/dL)	18.00–45.00	31.13
CREA (mg/dL)	0.08–0.13	0.10
ALT (U/L)	27.00–78.00	63.00
ALB (g/L)	26.57-–35.43	29.70
Ca (mmol/L)	2.18–2.44	2.46
IP (mg/dL)	5.53–10.19	9.71

## Supplementary Materials

Supplementary materials can be found at https://www.mdpi.com/1422-0067/20/5/1148/s1.
