# Supplementary material for: Rare-Earth-Doped Calcium Carbonate Exposed to X-ray Irradiation to Induce Reactive Oxygen Species for Tumor Treatment
Source: Int J Mol Sci. 2019 Mar 6;20(5):1148. doi: 10.3390/ijms20051148 (PMC6429163; doi:10.3390/ijms20051148)
Supplement: Supplementary file 1 [file ijms-20-01148-s001.pdf]

Rare-earth-doped Calcium Carbonate exposed to X-ray to induce Reactive Oxygen Species for Tumor Treatment

Chun-Chen Yang[a], Wei-Yun Wang[a], Feng-Huei Lin[b, c,*] , Chun-Han Hou[d,*]

[a] Department of Materials Science and Engineering, National Taiwan University, Taipei 10617, Taiwan

[b] Institute of Biomedical Engineering, National Taiwan University, Taipei 10617, Taiwan

[c] Institute of Biomedical Engineering and Nanomedicine, National Health Research Institutes, Miaoli 35053, Taiwan

[d] Department of Orthopedic Surgery, National Taiwan University, Taipei 10617, Taiwan

Corresponding Author

* Chun-Han Hou: phone, +886-2-23123456#65274; fax, +886-2-23971266; Email, chhou@ntu.edu.tw.

* Feng-Huei Lin: phone, +886-2-27327474; fax, +886-2-27327474; Email, double@ntu.edu.tw.

Table S1. $CaCO_3$:Ce measured by BET.

$CaCO_3$:Ce	BET Surface Area：<br>35.8875±0.1818 m^2/g<br>Correlation Coefficient: 0.9999237<br>Langmuir Surface Area: 48.6989±0.6808 m^2/g

	Correlation Coefficient: 0.9996093<br><br>BJH Adsorption Average Pore Diameter ：<br><br>1295.3808 Å<br><br>BJH Desorption Average Pore Diameter：<br><br>1228.4492Å
